# Supplementary material for: A single-cell atlas of conventional central chondrosarcoma reveals the role of endoplasmic reticulum stress in malignant transformation
Source: Commun Biol. 2024 Jan 24;7:124. doi: 10.1038/s42003-024-05790-w (PMC10808239; doi:10.1038/s42003-024-05790-w)
Supplement: Supplementary file 2 — Supplementary Information [file 42003_2024_5790_MOESM2_ESM.pdf]

communications biology

Supplementary Information

A single-cell atlas of conventional central chondrosarcoma reveals the role of endoplasmic reticulum stress in malignant transformation

Zezhuo Su et. al.

communications biology

a	Ben	Low_1	Low_2	Med	High_1	High_2	COS
Type of tumor	enchondroma	conventional chondrosarcoma	conventional chondrosarcoma	conventional chondrosarcoma	conventional chondrosarcoma	conventional chondrosarcoma	chondroblastic osteosarcoma
Histology	benign	low grade/G1	low grade/G1	medium grade/G2	high grade/G3	high grade/G3	high grade/G3
Gender	male	female	male	male	male	male	male
Age	38	76	16	50	68	52	39
Anatomical site	finger	femur	os calcis	pelvis	femur	thigh	femur
Number of Cells	5261	2325	6489	4216	4103	5129	4557
Mean Reads per Cell	96807	219794	66687	108489	139513	161499	111770
Median Genes per Cell	2320	3367	2301	2348	2493	2397	3285

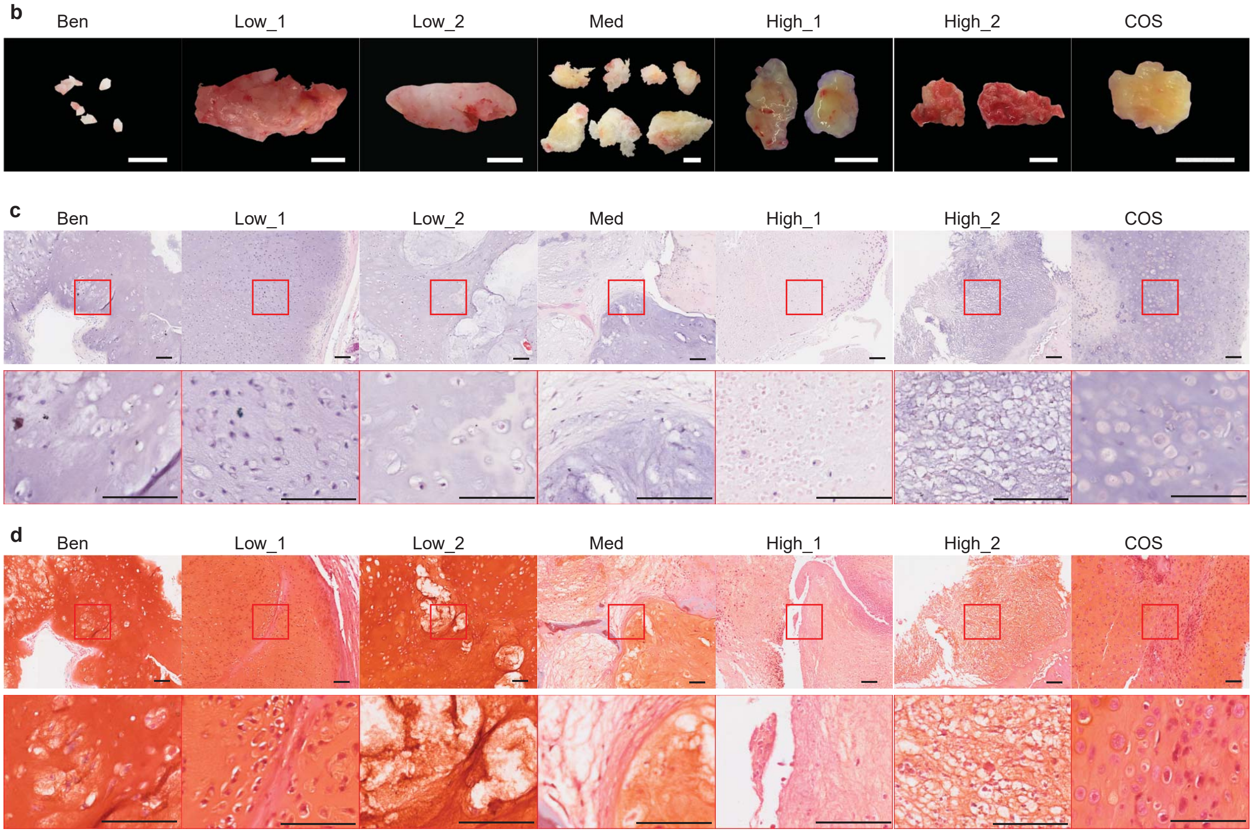

Supplementary Fig. 1 Pathological diagnosis and summary of scRNA-seq of tumour specimens. a. Diagnosis and Cell Ranger summary of tumour specimens. **b.** Images showing the macroscopic morphology of patient samples collected. Scale bar = 1cm. **c.** Haematoxylin and eosin staining illustrate the histological appearance of patient samples collected. Bottom panel shows the higher magnification section of the top panel. Scale bar = 100 μm. **d.** Safranin-O and fast green staining showing the presence of proteoglycan in the matrix of tumour specimens. Bottom panel shows the higher magnification section of top panel. Scale bar = 100 μm.

communications
biology

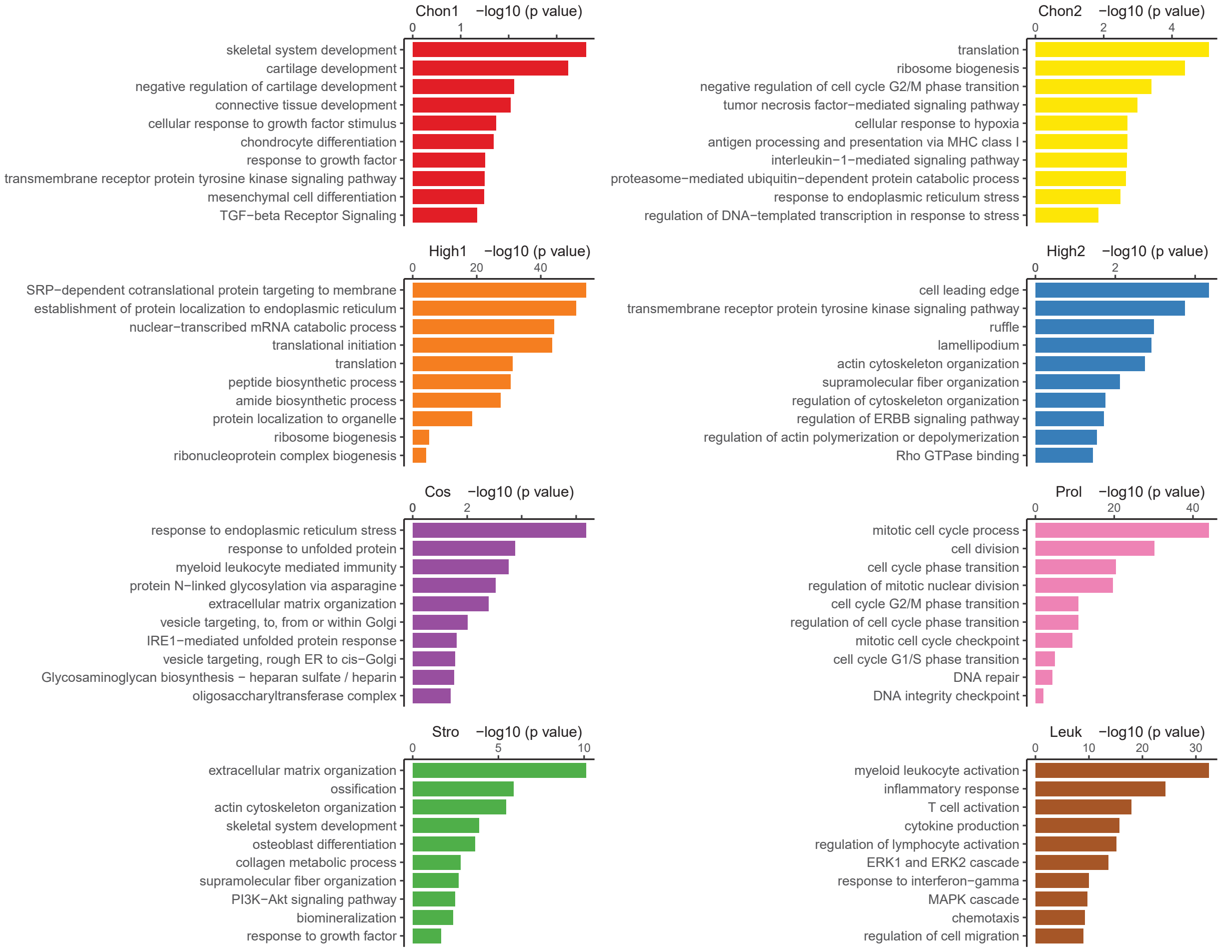

Supplementary Fig. 2. Characterization of distinct cell clusters found in conventional chondrosarcoma. Representative enriched gene ontology terms for each cell cluster.

communications
biology

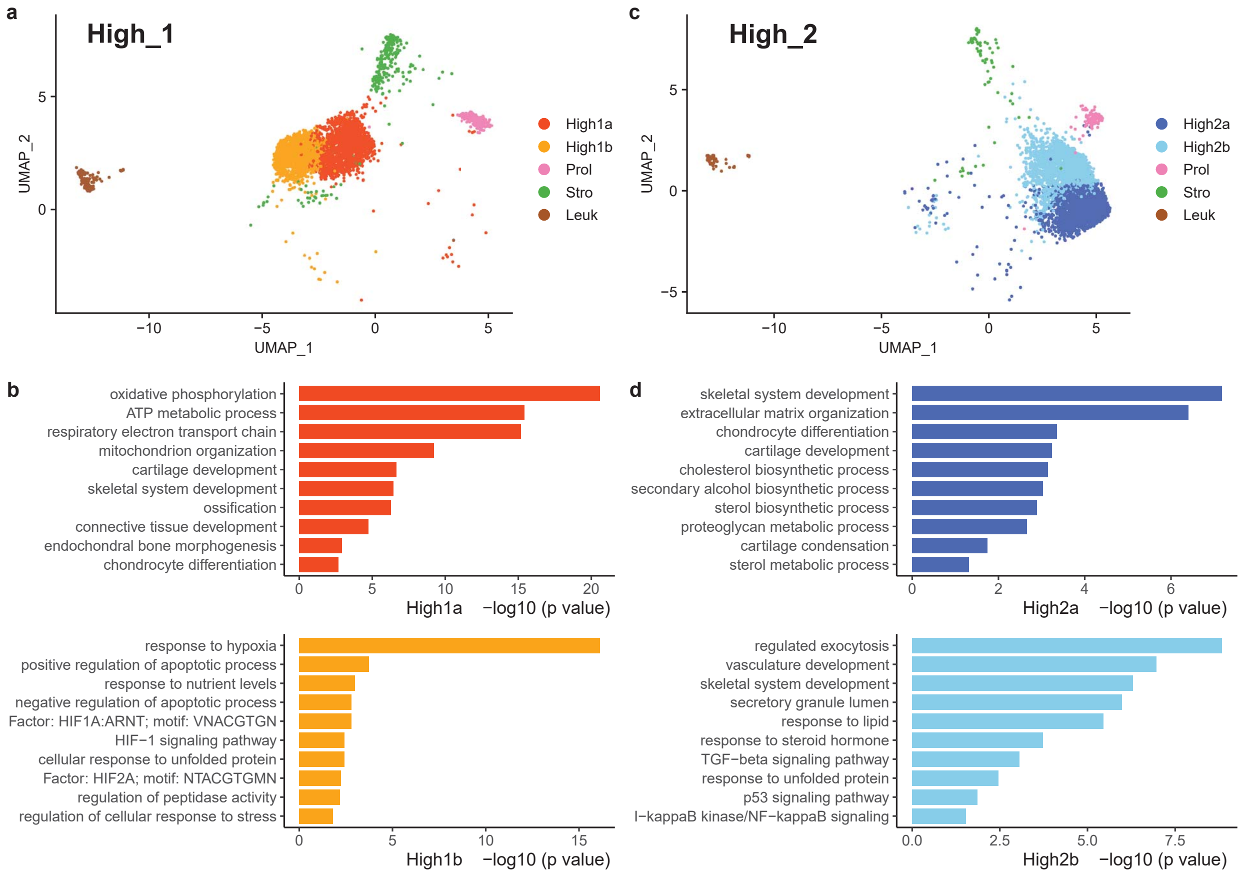

Supplementary Fig. 3 Characterization on the major neoplastic cell population in High_1 and High_2. a. The UMAP plot shows the subclusters of High1 cell cluster in High_1 . **b.** Gene ontology enrichment analysis on DEGs shows representative gene ontology terms enriched for High1 subclusters. **c.** The UMAP plot shows the subclusters of High2 cell cluster in High_2. **d.** Gene ontology enrichment analysis on DEGs shows representative gene ontology and pathway terms enriched for High2 subclusters.

communications
biology

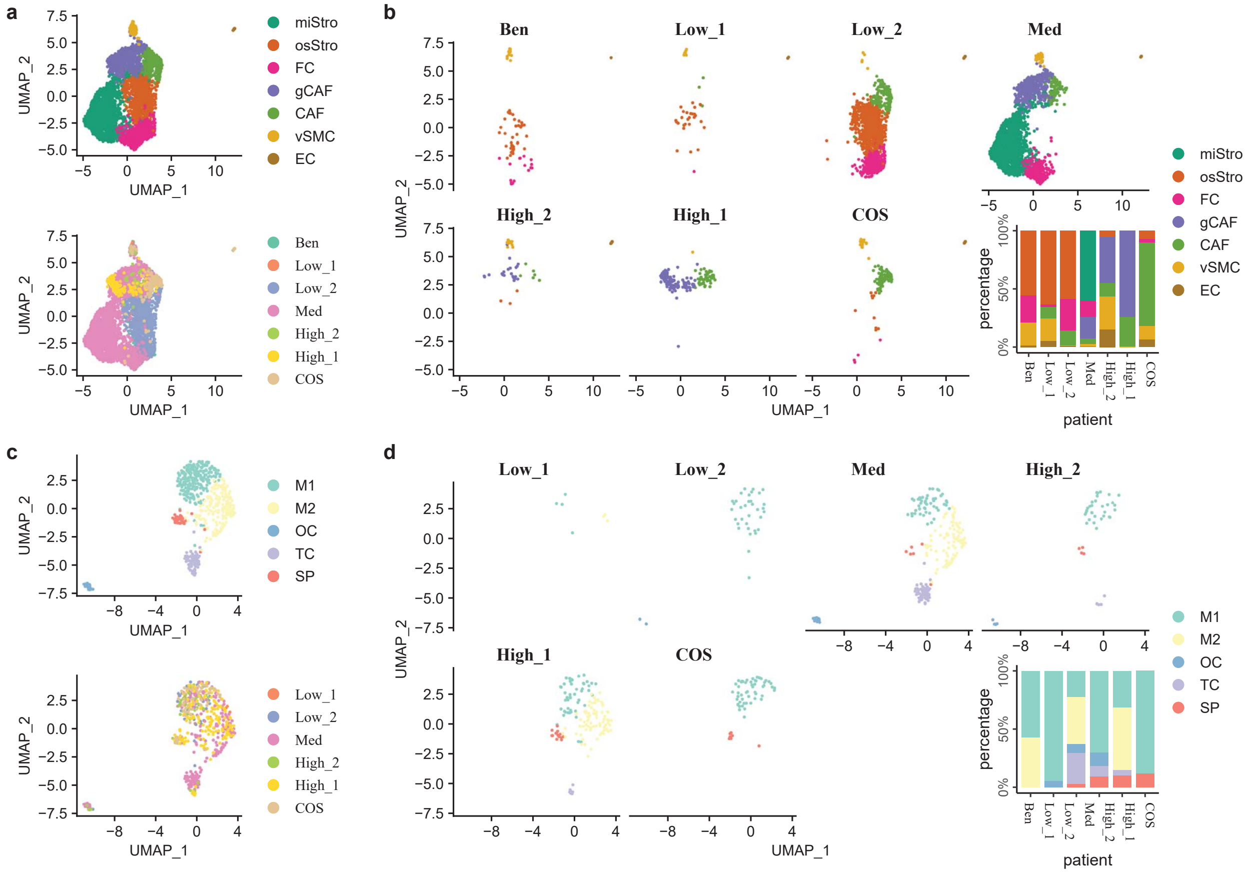

Supplementary Fig. 4 Heterogenous tumour microenvironment characterized by distinct stromal cell types. a. Representative UMAP plots of Stro cell cluster illustrate the identification of cell subclusters (top) and the origin of cell (bottom). **b.** The UMAP plot of Stro cell cluster split by patient illustrates the cell subclusters and a bar chart shows the abundance of subclusters in each patient. **c.** Representative UMAP plots of Leuk cell cluster illustrate the identification of cell subclusters (top) and the origin of cell (bottom). **d.** The UMAP plot of Leuk cell cluster split by patient illustrates the cell subclusters and a bar chart shows the abundance of subclusters in each patient. miStro, mineralization Stromal cell. osStro, ossification Stromal cell. FC, Fibrocartilage Chondrocyte. gCAF, granulating Cancer Associated Fibroblast. CAF, Cancer Associated Fibroblast. vSMC, vascular Smooth Muscle Cell. EC, Endothelial Cell. M1, M1 macrophage. M2, M2 macrophage. OC, Osteoclast. TC, T Cell. SP, Skeletal Progenitor.

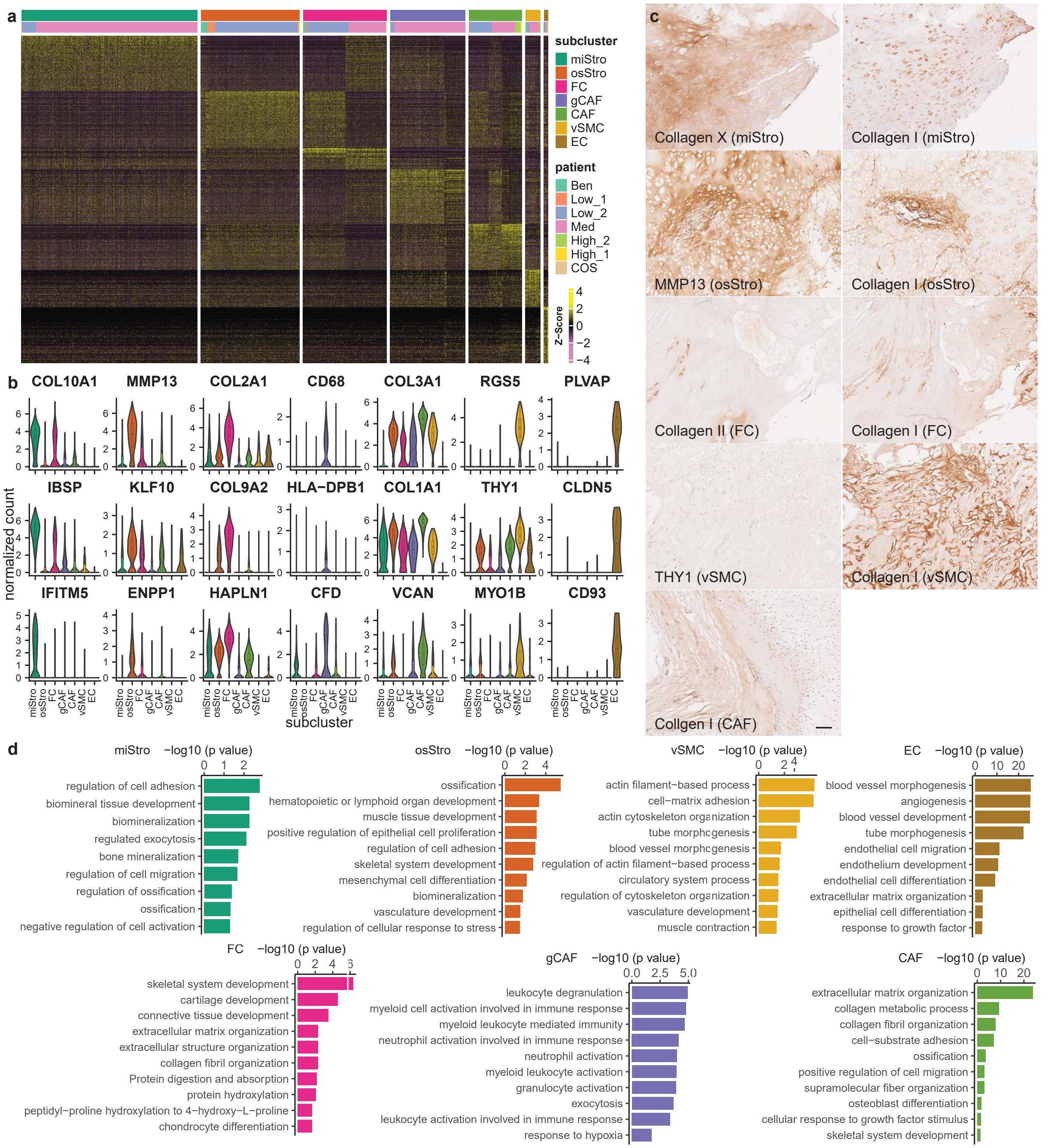

Supplementary Fig. 5 Distinct stromal cell types in Stro cell cluster. a. The heatmap shows the expression level of all specific marker genes in each Stro subcluster. **b.** Violin plots display the expression of representative marker genes across the subclusters of Stro. *COL1A1* is expressed in all cell types of Stro except EC. CAF and vSMC have highest expression level of *COL1A1* and *THY1*, respectively. **c.** Immunohistochemistry shows representative cluster marker genes *in situ*. Scale bar = 100 µm. **d.** Representative enriched gene ontology terms of each Stro subcluster. Box plots show the median, first and third quartiles, and minimum and maximum values within 1.5 times interquartile range.

communications
biology

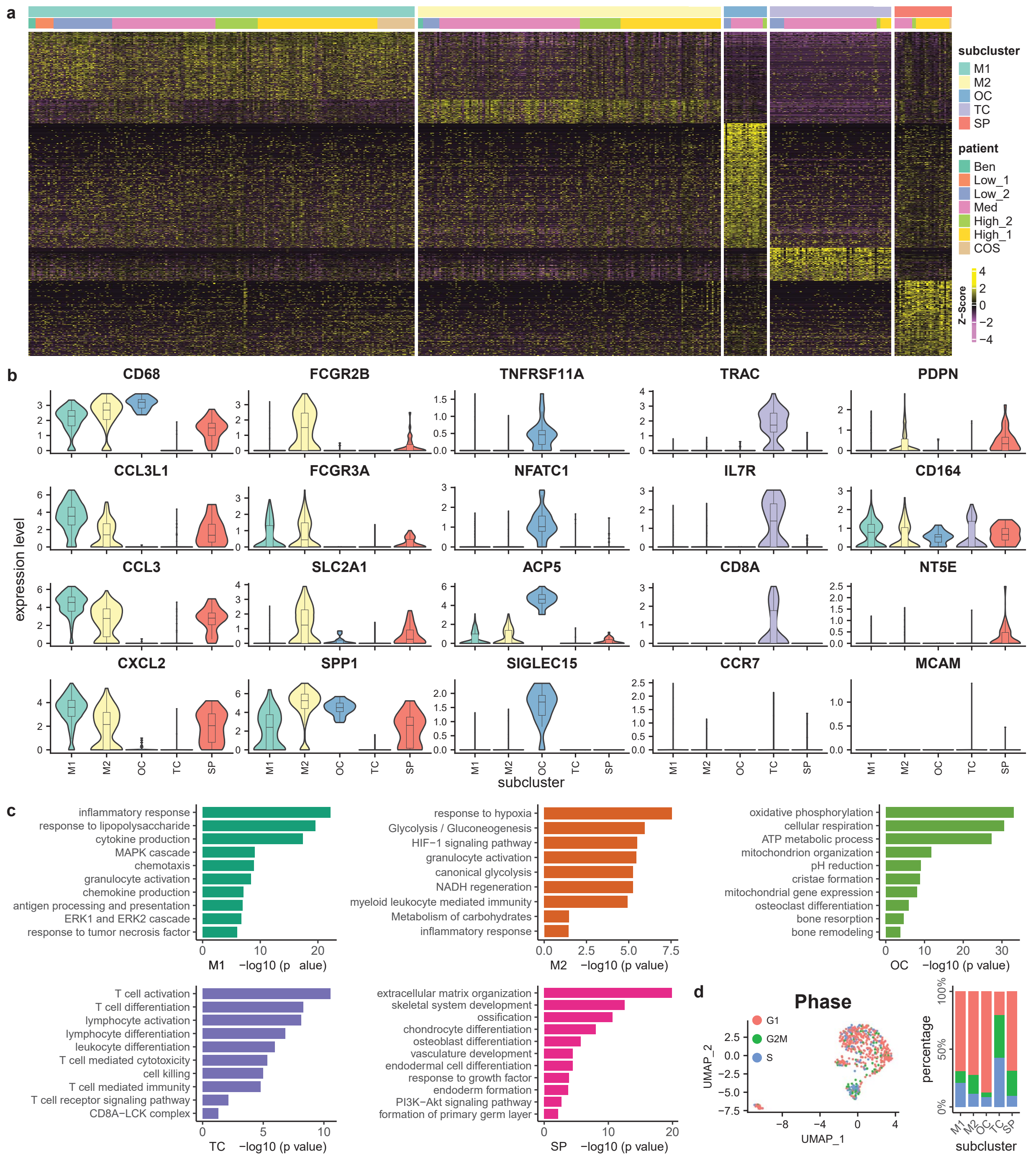

Supplementary Fig. 6 Distinct stromal cell types in Leuk cell cluster. a. The heatmap shows expression level of all specific marker genes in each Leuk subcluster. **b.** Violin plots displays the expression of representative genes across the subclusters in Leuk. *CCR7*, *CD164*, and *MCAM* are not cell−type−specific markers. **c.** Representative enriched gene ontology terms of each Leuk subcluster. **d.** The UMAP shows the cell cycle phases of each cell. The bar chart illustrates the frequency of G1, S, and G2M cell cycle phase of each Leuk subcluster, respectively. Box plots show the median, first and third quartiles, and minimum and maximum values within 1.5 times interquartile range.

communications biology

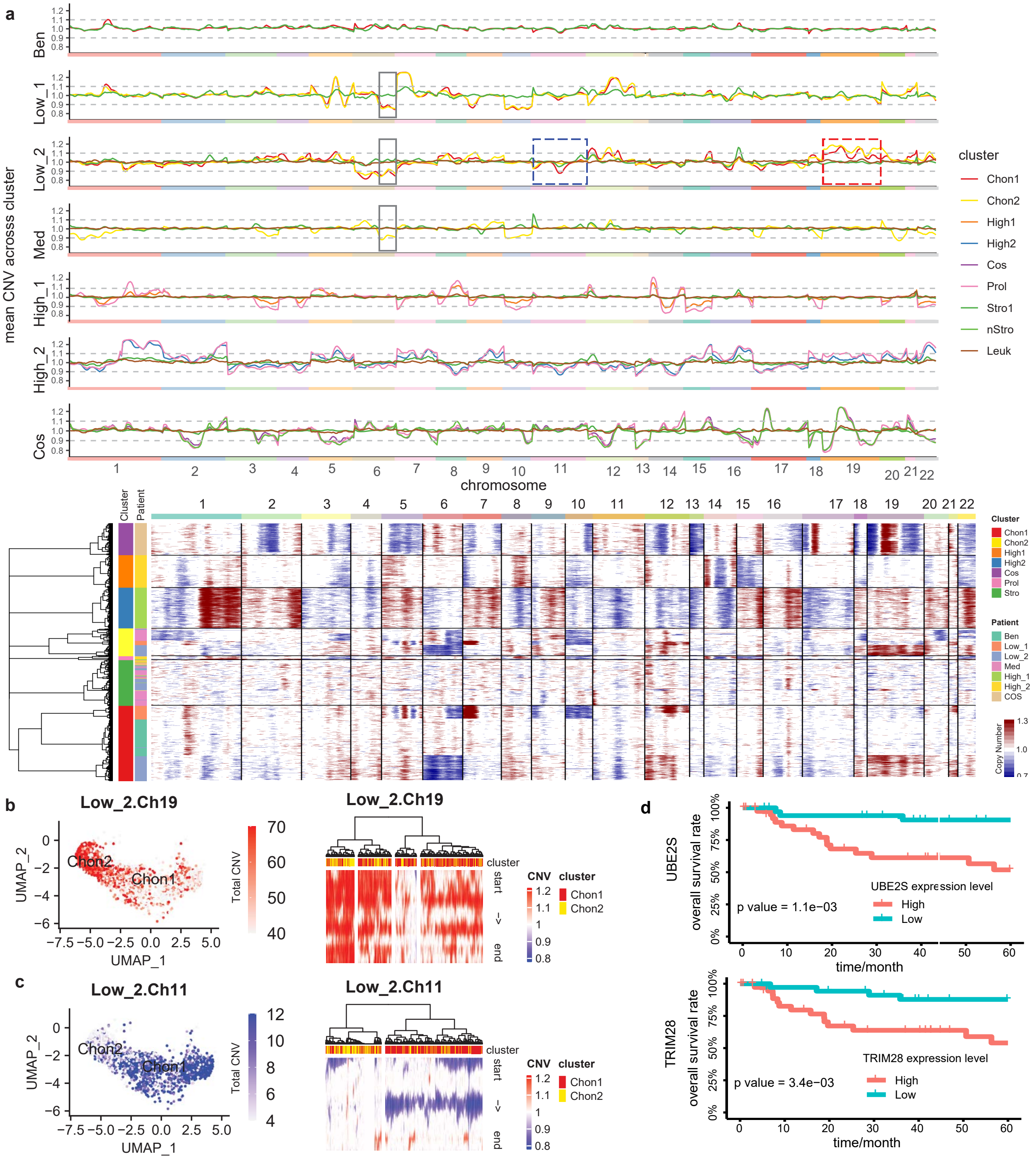

Supplementary Fig. 7 Copy number variation (CNV) heterogeneity of individual patients. **a.** Line plots (top) show the mean CNV across clusters in each patient. The y axis shows the mean copy number. The x axis shows the chromosome from 1 to 22. The heatmap (bottom) displays CNV of individual cells. **b.** CNV of chromosome 19 in Low_2. The feature plot displays total copy number of Chon1 and Chon2 cells. The heatmap shows the CNV of individual Chon1 and Chon2 cells. Hierarchical clustering was performed using "ward.D2" method. **c.** CNV of chromosome 11 in Low_2. The feature plot displays total copy number of Chon1 and Chon2 cells. The heatmap shows the CNV of individual Chon1 and Chon2 cells. Hierarchical clustering was performed using "ward.D2" method. **d.** Overall survival cures of patients stratified by the expression of UBE2S or TRIM28.

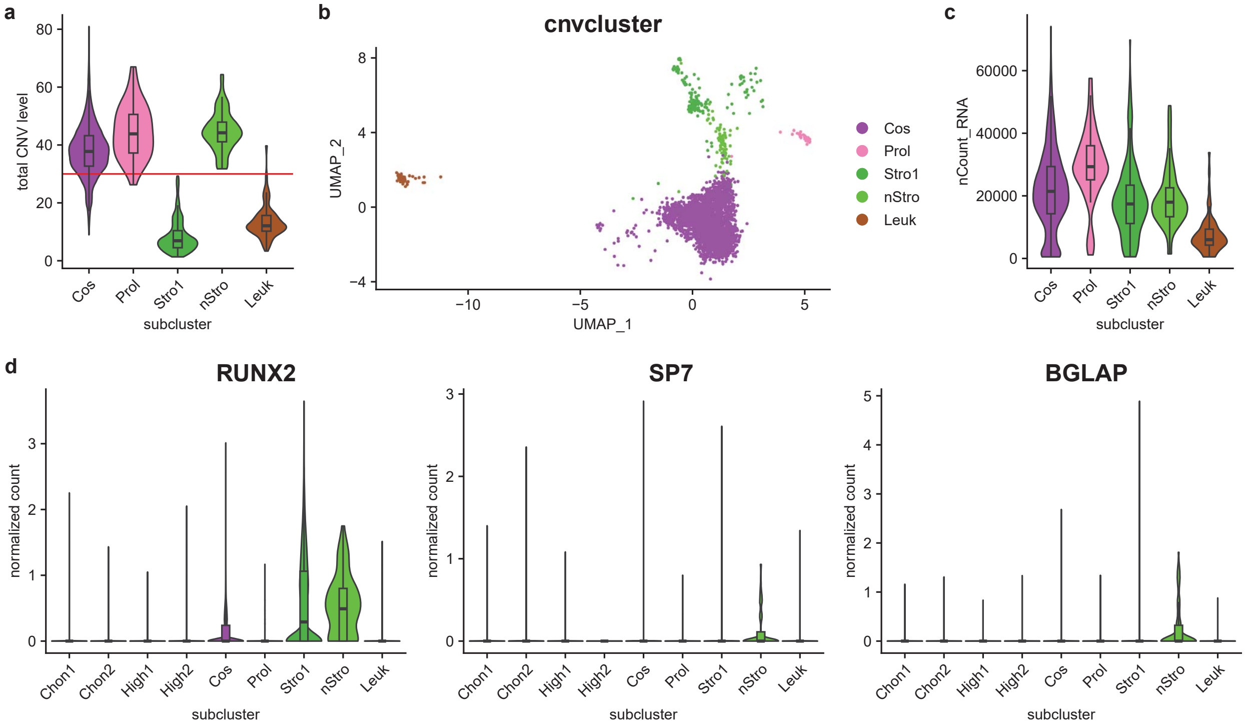

Supplementary Fig. 8. Characterization on the neoplastic cell population in chondroblastic osteosarcoma. a. The violin plot showing total CNV level of cells in the chondroblastic osteosarcoma (COS) grouped by subclusters. The stromal cells comprise of two cell populations with distinct total CNV. nStro, neoplastic stromal cells (total CNV > 30). Stro1, stromal cells with total CNV < 30. **b.** The UMAP plot showing the cell type composition of the COS. **c.** The violin plot showing the total count of RNA of individual cells across cell clusters. **d.** Violin plots showing expression of representative osteogenic genes across subclusters. Box plots show the median, first and third quartiles, and minimum and maximum values within 1.5 times interquartile range.

communications
biology

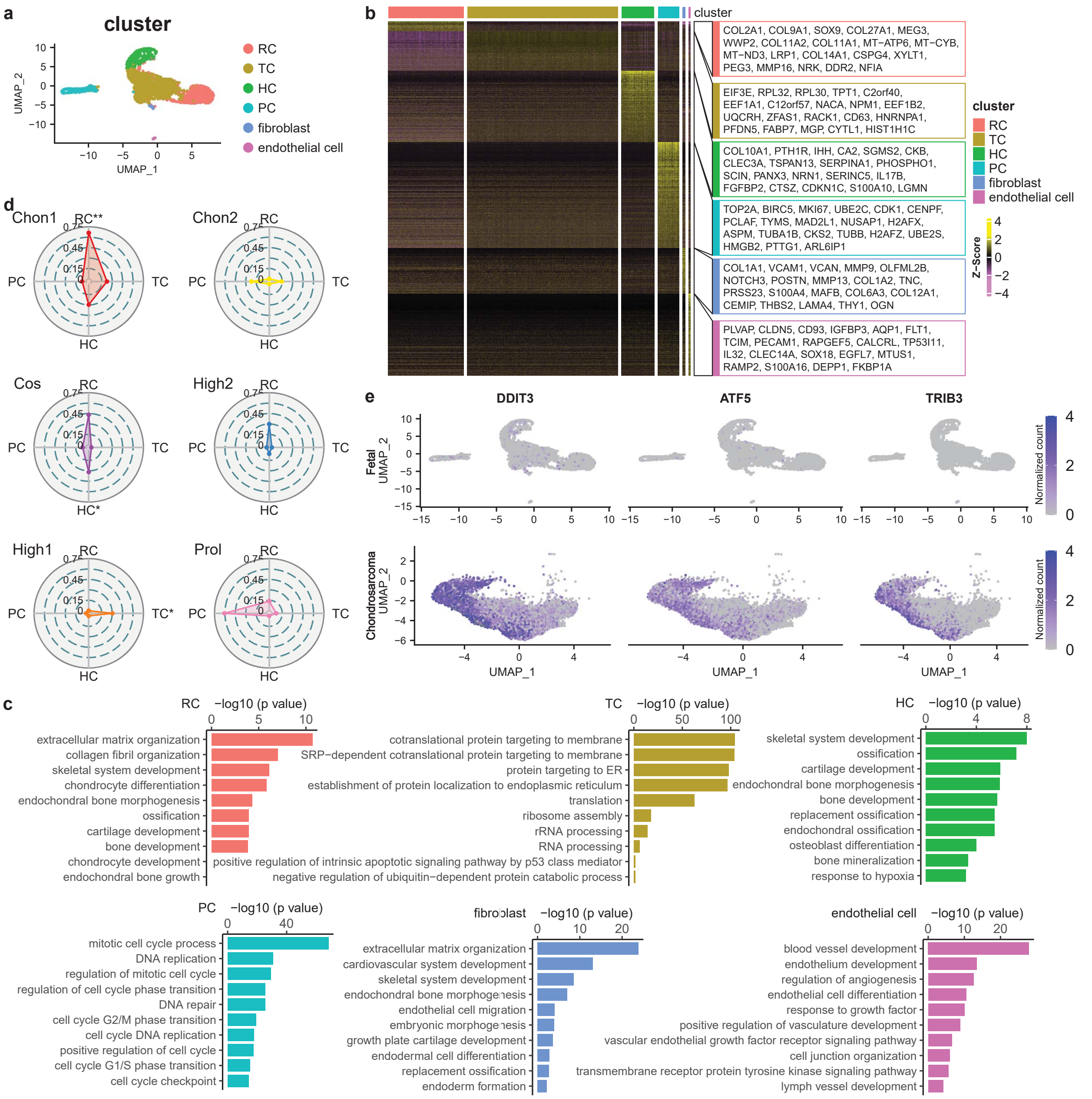

Supplementary Fig. 9 Anchoring Chon1 and Chon2 cells with foetal femur chondrocytes by canonical correlation analysis. a. Representative UMAP plots illustrate the identification of cell types from the foetal femur. **b.** The heatmap shows the expression level of all specific marker genes of each cell types from the foetal femur. Representative marker genes are highlighted in the right text box. **c.** Representative enriched gene ontology terms of each cluster in foetal femur. **d.** The radar plot shows the canonical correlation analysis (CCA) coefficients between different types of foetal chondrocyte and Chon1 and Chon2 cells. *, p value < 0.5. **, p value < 0.01 by permutation test. **e.** Feature plots show the expression of *DDIT3*, *ATF5*, and *TRIB3* in the foetal femur chondrocytes (top) and Chon1 and Chon2 cells (bottom), respectively. RC, Resting Chondrocyte. TC, Transitioning Chondrocyte. HC, hypertrophic Chondrocyte. PC, Proliferating Chondrocytes.

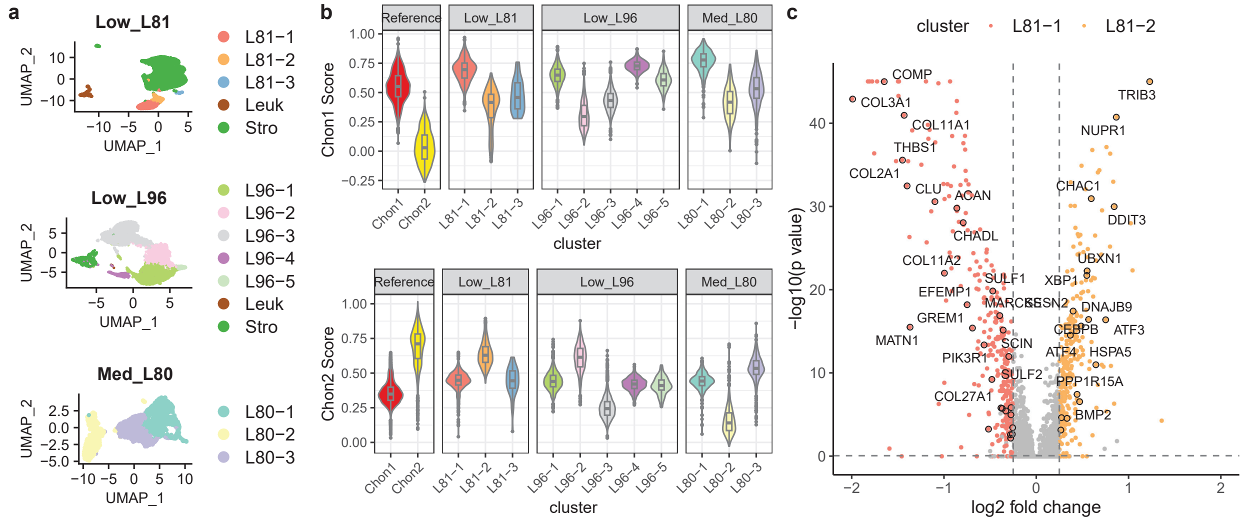

Supplementary Fig. 10 Chon2 cluster featured by response to ER stress is a marker for conventional chondrosarcoma. a. UMAP plots showing the identification of cell clusters in a new batch of samples (i.e., Low_L81, Low_L96, and Med_L80). **b.** Violin plots showing Chon1 and Chon2 cluster scores of Chon2 and Chon1 clusters from differentiated tumours as a reference and individual malignant clusters of the new batch of samples. **c.** Differential expressed genes between L81-1 and L81-2. Genes enriched in chondrocyte differentiation (GO:0002062) and response to endoplasmic reticulum stress (GO:0034976) gene ontology terms are labelled. Box plots show the median, first and third quartiles, and minimum and maximum values within 1.5 times interquartile range.

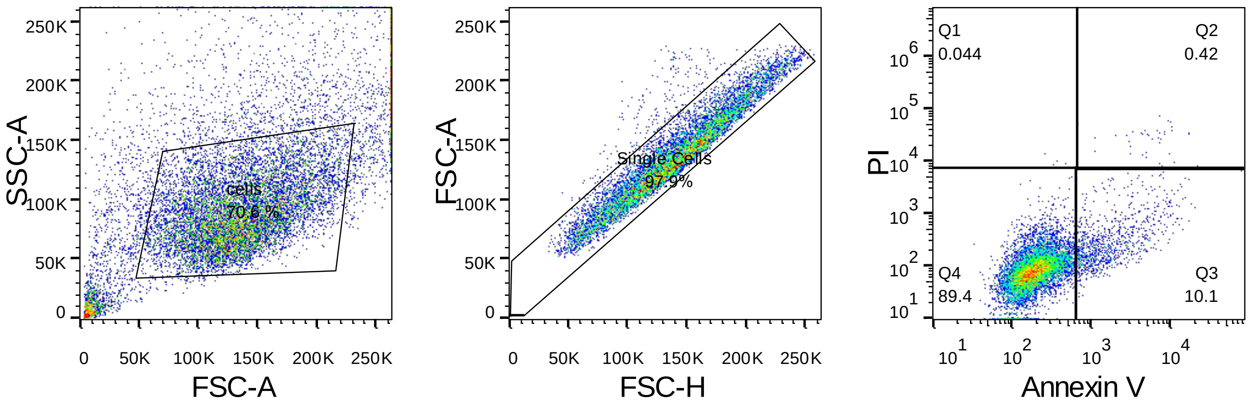

Supplementary Fig. 11 Gating strategy of flow cytometry analysis of apoptosis.

communications
biology

a

	benign	low	medium	high	dedifferentiated
Ben_bulk	2.564e−03	4.766e−01	1.000e+00	1.810e−01	6.471e−02
Med_bulk	3.385e−01	5.080e−01	1.659e−02	5.053e−01	7.256e−01
High_bulk	5.915e−01	4.481e−01	3.784e−01	2.621e−05	1.174e−01
Ded_bulk	5.434e−01	3.477e−01	8.989e−03	1.000e+00	8.505e−06
Stro_bulk	4.179e−01	7.749e−02	8.191e−01	2.451e−01	1.000e+00

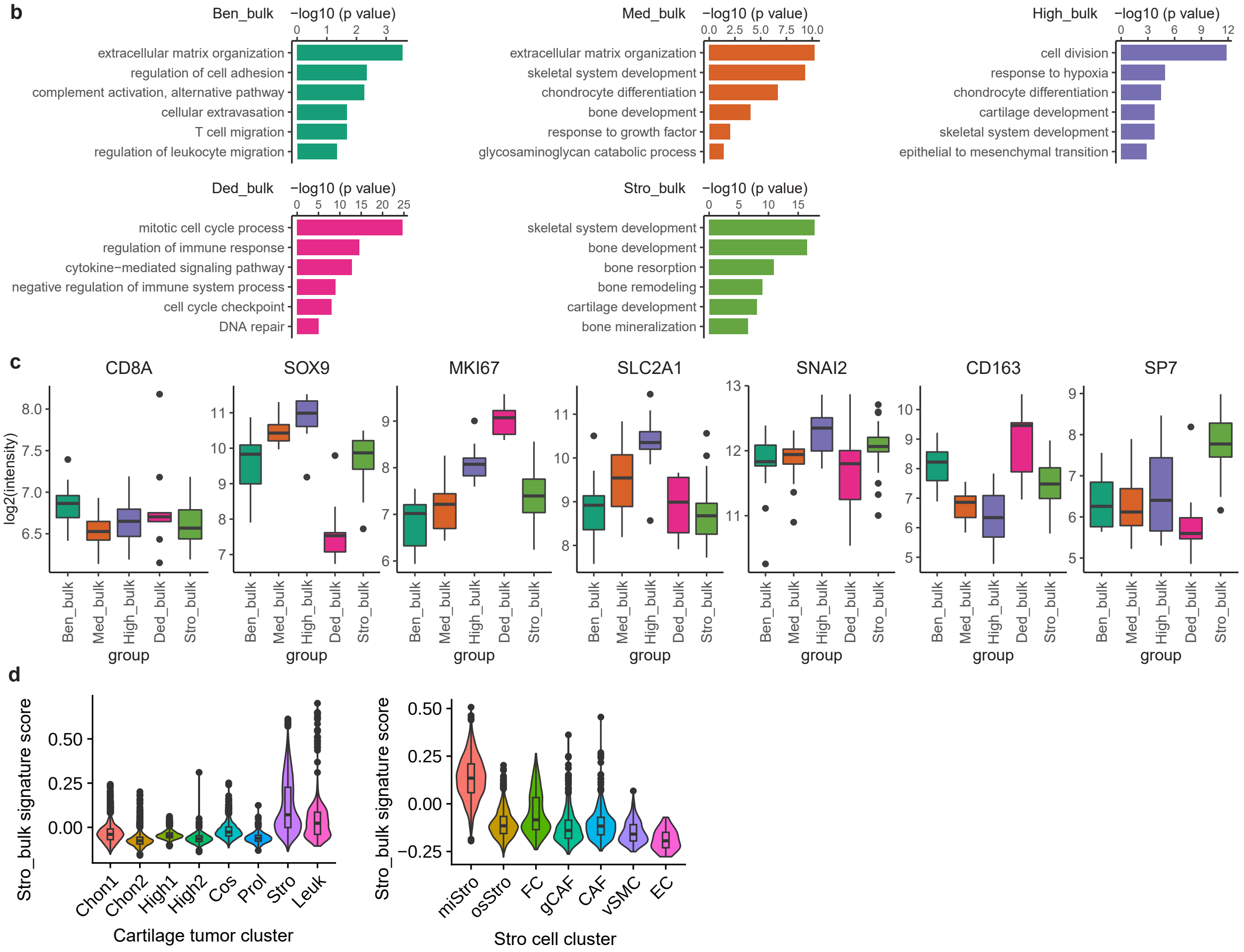

Supplementary Fig. 12. Molecular characterization of bulk expression groups of cartilage tumours determined by single-cell signatures. a. Fisher's exact test shows the correlation between bulk-mRAN-based groups and histology grades. **b.** Representative gene ontology of each bulk-mRNA-based group. **c.** The box plots displaying the expression intensity of representative genes across bulk groups. **d.** The Stro_bulk signature score in cartilage tumor clusters and Stro cell clusters, respectively. Box plots show the median, first and third quartiles, and minimum and maximum values within 1.5 times interquartile range.

communications
biology

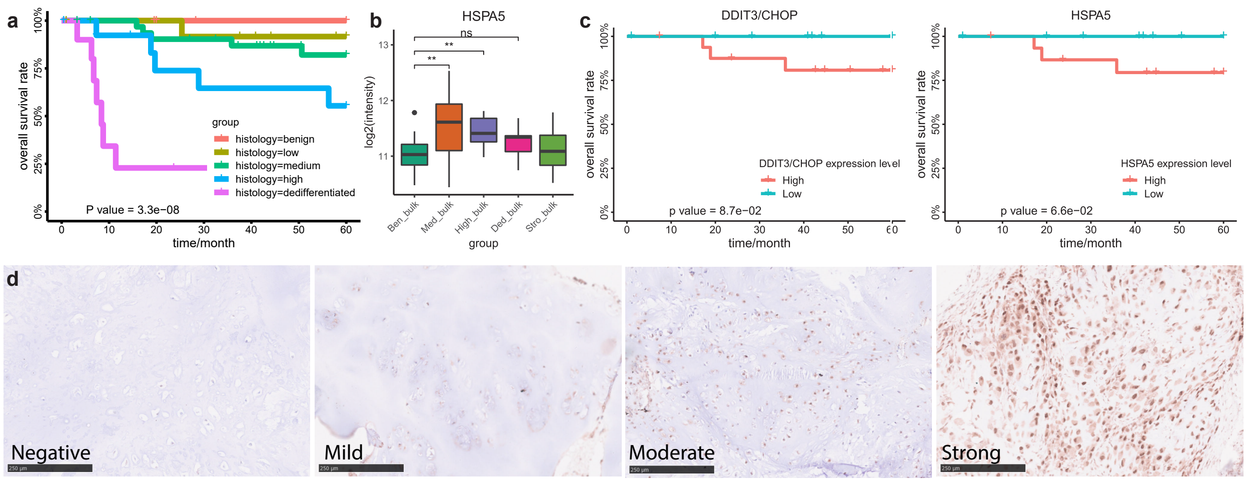

Supplementary Fig. 13. ER stress markers for the diagnosis of chondrosarcoma. a. Overall survival rates of patients from individual histological groups estimated using Kaplan−Meier analysis. **b.** The box plots displaying expression intensity of *HSPA5* across bulk groups. **c.** Overall survival cures of patients from Ben_bulk or Med_bulk group. Patients are classified into high and low groups according to the expression of *DDIT3* or *HSPA5*. **d.** Representative images show DDIT3/CHOP immunohistochemistry staining with negative (0-1), mild (2-3), moderate (4-8), and strong (9-12) immunoreactive scores. Scale bar = 250 µm. Box plots show the median, first and third quartiles, and minimum and maximum values within 1.5 times interquartile range. P values by Student's T test are shown in the plot. ns, not significant, *, p value < 0.5. **, p value < 0.01.
