# Supplementary material for: A single-cell atlas of conventional central chondrosarcoma reveals the role of endoplasmic reticulum stress in malignant transformation
Source: Commun Biol. 2024 Jan 24;7:124. doi: 10.1038/s42003-024-05790-w (PMC10808239; doi:10.1038/s42003-024-05790-w)
Supplement: Supplementary file 3 — Description of Additional Supplementary Files [file 42003_2024_5790_MOESM3_ESM.pdf]

Description of Additional Supplementary Files

File name: Supplementary Data 1
Description: Copy number variation.

File name: Supplementary Data 2
Description: Comparison with osteoarthritis.

File name: Supplementary Data 3
Description: The source data of gene markers behind the graphs in the paper.

File name: Supplementary Data 4
Description: The source data of batch 2 samples behind the graphs in the paper.

File name: Supplementary Data 5
Description: The source data of enrichment analysis behind the graphs in the paper.

File name: Supplementary Data 6
Description: The source data for main figures.
